# Structural and Functional Deficits in a Neuronal Calcium Sensor-1 Mutant Identified in a Case of Autistic Spectrum Disorder

**DOI:** 10.1371/journal.pone.0010534

**Published:** 2010-05-07

**Authors:** Mark T. W. Handley, Lu-Yun Lian, Lee P. Haynes, Robert D. Burgoyne

**Affiliations:** 1 The Physiological Laboratory, School of Biomedical Sciences, University of Liverpool, Liverpool, United Kingdom; 2 School of Biological Sciences, University of Liverpool, Liverpool, United Kingdom; University of Oldenburg, Germany

## Abstract

Neuronal calcium sensor-1 (NCS-1) is a Ca^2+^ sensor protein that has been implicated in the regulation of various aspects of neuronal development and neurotransmission. It exerts its effects through interactions with a range of target proteins one of which is interleukin receptor accessory protein like-1 (IL1RAPL1) protein. Mutations in IL1RAPL1 have recently been associated with autism spectrum disorders and a missense mutation (R102Q) on NCS-1 has been found in one individual with autism. We have examined the effect of this mutation on the structure and function of NCS-1. From use of NMR spectroscopy, it appeared that the R102Q affected the structure of the protein particularly with an increase in the extent of conformational exchange in the C-terminus of the protein. Despite this change NCS-1(R102Q) did not show changes in its affinity for Ca^2+^ or binding to IL1RAPL1 and its intracellular localisation was unaffected. Assessment of NCS-1 dynamics indicated that it could rapidly cycle between cytosolic and membrane pools and that the cycling onto the plasma membrane was specifically changed in NCS-1(R102Q) with the loss of a Ca^2+^ -dependent component. From these data we speculate that impairment of the normal cycling of NCS-1 by the R102Q mutation could have subtle effects on neuronal signalling and physiology in the developing and adult brain.

## Introduction

Calcium has effects on multiple aspects of neuronal function through the action of Ca^2+^-sensor proteins. The Neuronal Calcium Sensor (NCS) proteins are a family of such proteins that are predominantly expressed in neurones and are characterised by their structural similarities and a high (low micromolar) affinity for Ca^2+^ [1]. Each NCS protein contains four EF-hand Ca^2+^-binding domains of which 2–3 have the capacity to bind Ca^2+^, and many are N-terminally myristoylated [2]. Different NCS proteins can regulate distinct target proteins, and they can have highly specific, or more wide-ranging, distributions and cellular roles [1].

NCS-1 is the most conserved of the NCS protein family, with an orthologue, Frq1, found in yeast [3]. In multicellular organisms, it has multiple binding partners [4] and is involved in a range of cellular processes, though in broad terms, it has been most associated with central nervous system development and with neurotransmission. A role in development may be inferred from its regulation of neurite outgrowth and synapse formation, which has been seen in flies, molluscs, birds and mammals [5], [6], [7], [8], [9], [10], [11]. The likely importance of NCS-1 in complex and highly specialised developmental programs is suggested by studies showing that its transcription is under the control of homeobox and patterning genes in C. elegans, and that its expression is required for formation of the semicircular canals in zebrafish [12], [13], [14]. The involvement of NCS-1 in neurotransmission has been characterised in several systems [15], [16]. A recent knockout study in flies indicates that it is important in regulating presynaptic calcium-influx [17] and its over-expression has been shown to enhance short term potentiation [18], its expression is increased in long term potentiation [19], [20], and it is thought to be necessary for at least one form of long term depression [21]. Consistent with the suggestion that its effects on synaptic plasticity are relevant to central nervous system function, associative learning and memory is disrupted when NCS-1 is absent in *C. elegans*, and enhanced when it is over-expressed [22]. Furthermore, selective moderate over-expression of NCS-1 in the dentate gyrus of mice has recently been found to promote rapid acquisition of spatial memory [23].

Amongst the proteins that interact with NCS-1 is interleukin receptor assessory protein like-1 (IL1RAPL1) [24]. This is a transmembrane protein found mostly in the brain, and in particular in the hippocampus [25]. The protein contains three extracellular immunoglobulin-like domains, an intracellular Toll/Interleukin receptor domain and a specific domain [24]. It was found that part of the specific domain can bind to NCS-1 and this interaction may mediate the down-regulation of N-type calcium channels [24], [26]. Like NCS-1, IL1RAPL1 has been implicated in nervous system development [27], and in particular in neurite outgrowth and differentiation [26], [28]. The gene encoding IL1RAPL1 is on the X chromosome, and has been found to be mutated in a number of cases of X-linked mental retardation [25], [29], [30], [31], [32], and polymorphisms in the gene have been associated with differences in human cognition [33]. In a recent study, linkage analysis has suggested an association between loss of function mutations in the gene encoding IL1RAPL1 and a number of cases of familial autism and autistic spectrum disorder (ASD) [34]. Females carrying a loss of function mutation in IL1RAPL1 were found to exhibit a spectrum of phenotypes ranging from no obvious impairment to autism with or without mild mental retardation. Males with such a mutation were found to have mild to severe autism, mental retardation or both. In the same study a screen was also carried out on a cohort of 142 patients with ASD and 190 control subjects for mutations in NCS-1. Exon 4 of NCS-1 was not found to be mutated in any of the control subjects, but a point mutation, NCS-1(R102Q), was identified in one individual with ASD [34]. It is not known, however, whether there is any causal relationship between this amino acid change and ASD or whether instead this represents a polymorphism in the Jewish Sefrade population to which the subject belonged.

In previous studies linking NCS-1 to disease states [35], it has been found that expression of NCS-1 is elevated in the prefrontal cortices of patients with schizophrenia and bipolar disorder [36], [37], and is up-regulated in injured neurones and in epilepsy [38], [39]. NCS-1 has, however, previously only been speculatively associated with MR and ASD on the basis of its interaction with IL1RAPL1 [26], [34]. Furthermore, it is unclear whether the R102Q mutation/polymorphism in NCS-1 found in a single individual would have any consequences for NCS-1 function. In this study, we present evidence for structural and functional deficits in NCS-1(R102Q) suggesting that this mutation could indeed have pathological consequences.

## Results

### NMR spectroscopy identifies a structural deficit in NCS-1(R102Q)

**Figure 1:**
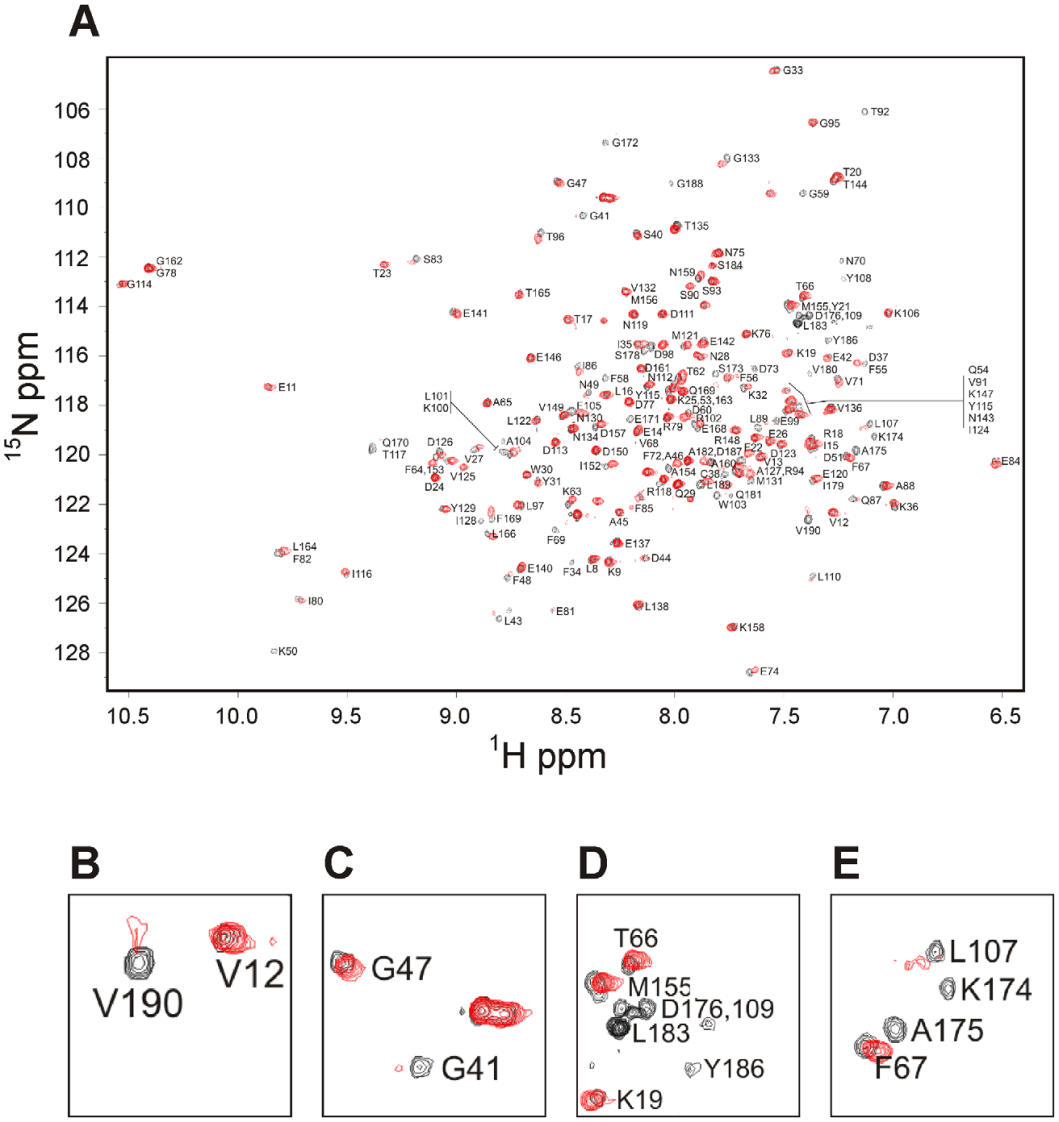
The R102 mutation modifes the ^1^H/^15^N HSQC spectrum of NCS-1. (A) Overlay of the ^1^H/^15^N HSQC spectra of wild type NCS-1 (black) and NCS-1(R102Q) (red). Residues assigned in the wild type NCS-1 are shown by residues number. (B–D) Examples of expanded regions of the overlaid spectra showing resonances that overlap, are shifted or absent in NCS-1(R102Q) compared to wild type protein.

On the basis of the published crystal structure of NCS-1 [40], the NCS-1(R102Q) mutation affects an exposed surface residue that forms part of an α-helix spanning residues 98–108 (helix F). Use of the AGADIR tool [41] predicted that this sequence has a high propensity to form a helix, in fact the highest compared to the rest of the NCS-1 polypeptide sequence. Interestingly the R102Q mutation reduced the α-helical propensity of surrounding sequence by approximately four-fold (Table S1). In order to determine whether this prediction was reflected in terms of altered protein structure, 2-D ^1^H/^15^N heteronuclear single quantum coherence (^1^H/^15^N HSQC) NMR spectra were acquired for the wild-type and mutant proteins. Unmyristoylated, ^15^N-labeled NCS-1 and NCS-1(R102Q) were prepared and spectra collected for the Ca^2+^ loaded samples. Resonance assignments of the ^1^H/^15^N HSQC spectrum of wild-type NCS-1 were made through comparison to published data [42] on unmyristoylated Ca^2+^ loaded NCS-1 (Biological Magnetic Resonance Data Bank accession number 4378). Resonances corresponding to all amino acid residues were assigned, with the exception of N-terminal residues 1–5, which are unstructured [42] (Figure 1A, black peaks). For NCS-1(R102Q), ^1^H/^15^N HSQC spectra resonances corresponding to the majority of residues coincided with those found in the spectrum of the wild type protein, indicating that most of the mutant is structurally identical to wild type protein (see Figure 1A, red). Strikingly however, a number of resonances were shifted or showed a dramatic reduction in intensity, and several were entirely absent (see examples in Figure 1, B–C and D–E respectively) indicating a substantial conformational exchange in the mutant protein.

**Figure 2:**
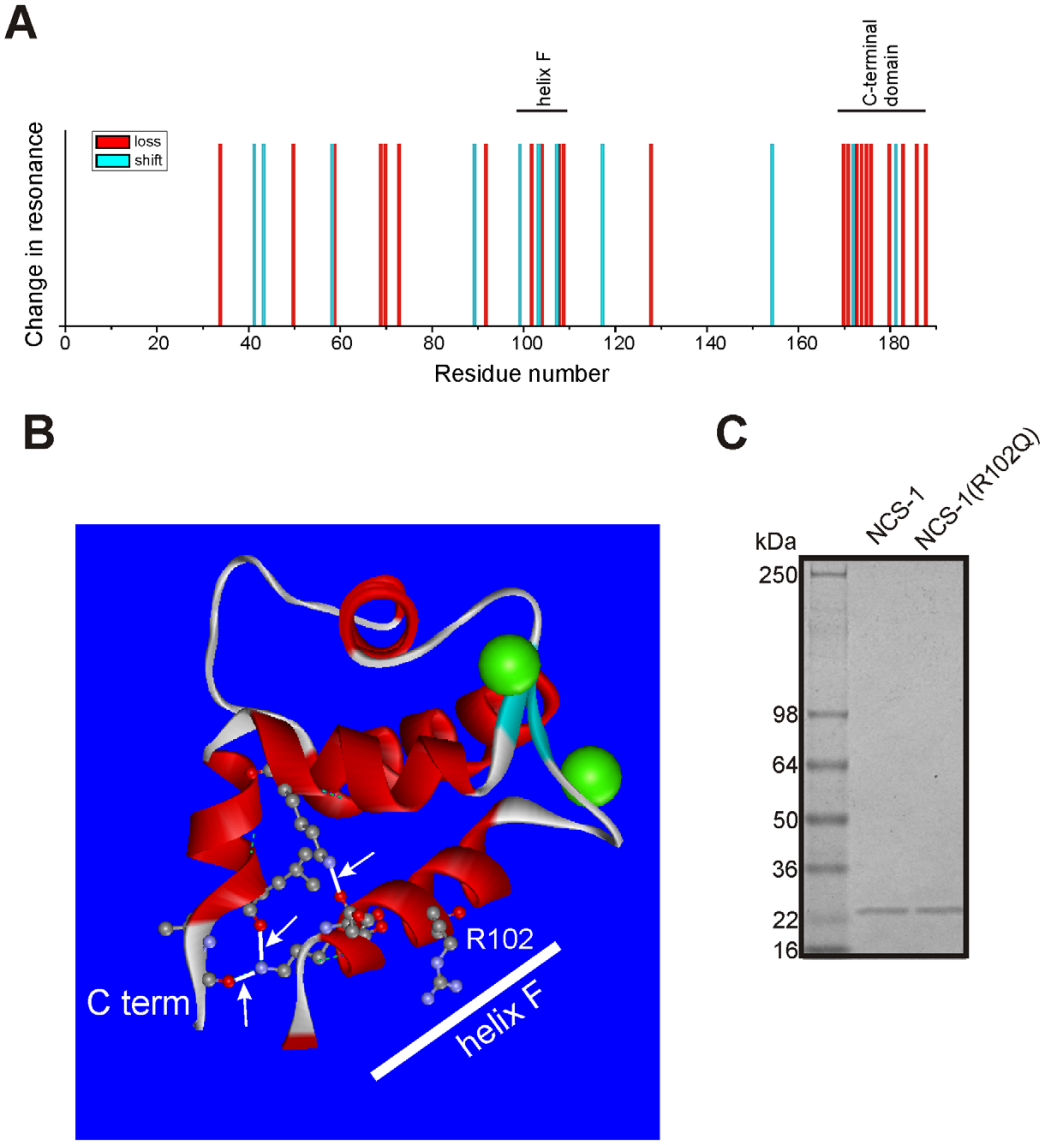
The C-terminus of NCS-1 is particularly affected by the R102Q mutation. (A) Distribution of resonances along the NCS-1 sequence that are the shifted or undetectable in the NCS-1(R102Q) ^1^H/^15^N HSQC spectrum. (B) Structure of the C-terminal half of NCS-1 (residues 93–186 rendered from the PDB file1G8I) showing the position of R102 within helix F and the hydrogen bond interactions between helix F and the C-terminal domain. (C) Coomassie blue-stained SDS-polyacrylamide gel showing the NCS-1 and NCS-1(R102Q) proteins after analysis by NMR spectroscopy.

Figure 2A shows a plot of the positions of shifted and ‘lost’ resonances from the ^1^H/^15^N HSQC spectra of NCS-1(R102Q) with respect to NCS-1 amino acid sequence. Resonance changes were seen throughout the polypeptide sequence with the number of changes more marked around the site of mutation in helix F (residues 99–109). Interestingly, many of the chemical shift perturbations also occurred at the C-terminus with the resonances of 10 of the C-terminal 21 residues being undetectable in the ^1^H/^15^N HSQC spectrum of NCS-1(R102Q). It appears that the R102Q mutation has given rise to long-range effects, leading to an increase in the dynamics of the C-terminus region. These residues correspond to a region in the crystal structure that is stabilised by hydrogen bonding [40] to helix F carrying the mutation. We speculate that the Arg to Gln mutation at position 102 perturbs the helical stability of helix F; this in turn disrupts the hydrogen-bonding network shown in Figure 2B, leading to the C-terminus region being structurally less constrained. The extreme line-broadening observed in the NMR spectrum for the C-terminus region is due to the resultant conformational exchange. It is possible to rule out C-terminus proteolysis since, firstly, peaks corresponding to some C-terminal residues remained (see Figure 1B), secondly, the recombinant protein samples used in the NMR spectroscopy were indistinguishable in size in the SDS –PAGE gels (see Figure 2C), and thirdly, mass spectrometry confirmed that both proteins were full-length with no evidence of truncations.

### NCS-1 and NCS-1(R102Q) possess comparable Ca^2+^-sensitivity in vitro

**Figure 3:**
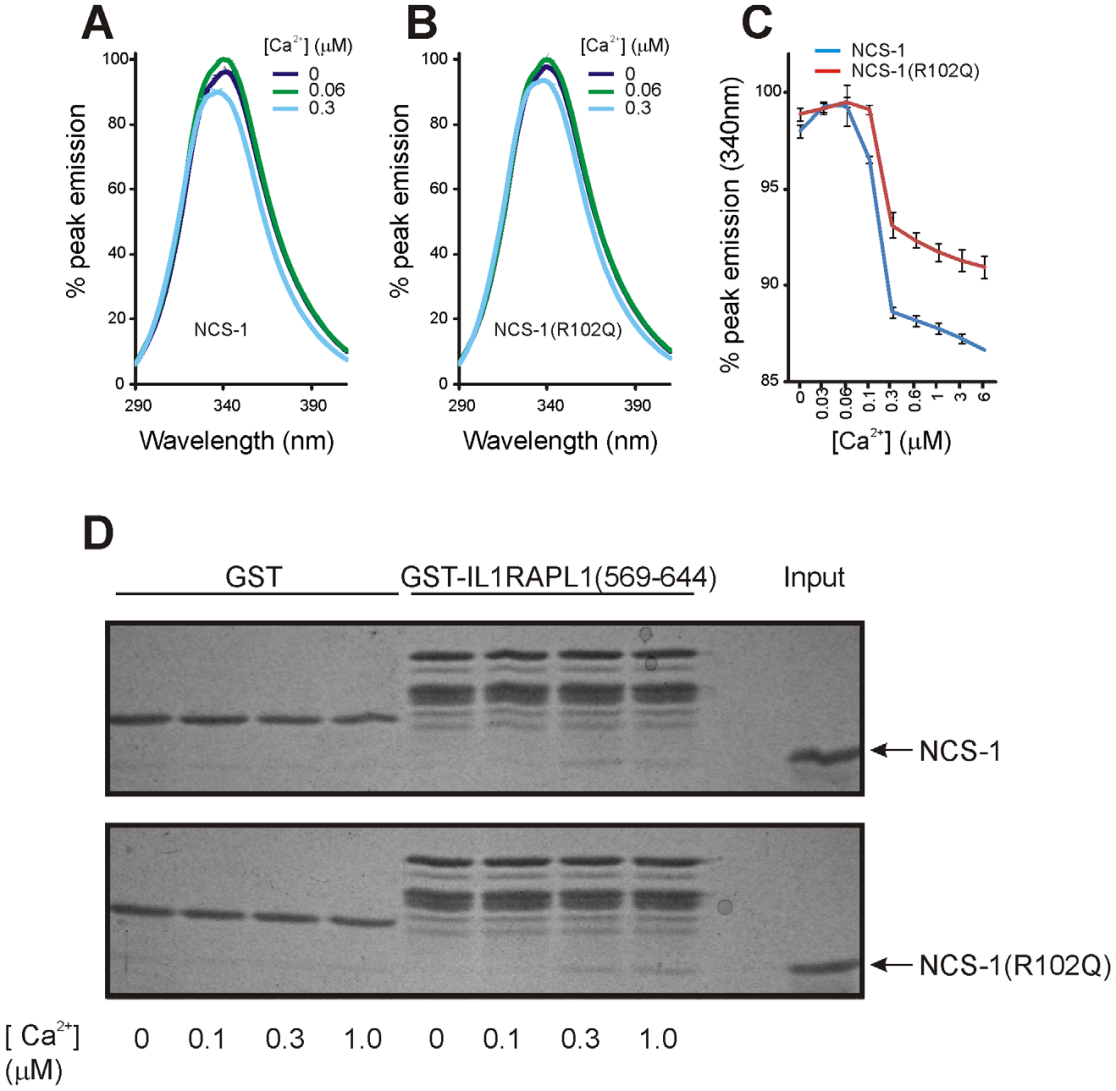
The R102Q mutation does not affect binding to IL1RAPL1 or Ca^2+^-binding. (A,B) The endogenous tryptophan fluorescence of NCS-1 and NCS-1(R102Q) was monitored by excitation at 280 nm and scanning over a range of emission wavelengths before and after step-wise addition of CaCl_2_ to give the indicated free Ca^2+^ concentrations. (C) The peak emission value for each emission trace was determined, normalised to the overall peak value for that experiment and the data expressed as the mean ± SEM (n = 10) for each free Ca^2+^ concentration tested. (D) NCS-1 and NCS-1(R102Q) were incubated with GST or GST-IL1RAPL1(569–644) in the absence or presence of the indicated free Ca^2+^ concentrations.

Having shown that the R102Q mutation affected the structure of NCS-1 we explored the possibility that it might affect the Ca^2+^-binding characteristics of the protein. We used changes in intrinsic tryptophan fluorescence to report the conformational change in NCS-1 and NCS-1(R102Q) in response to Ca^2+^ binding, as this approach has been successfully used previously [43], [44], [45], [46]. Proteins were excited with 280 nm light, and emission spectra from 290 nm to 410 nm were recorded as free Ca^2+^ concentrations in the buffer were incrementally increased. Saturating concentrations of free Mg^2+^ were present in the buffer to insure that this ion did not influence the results [44]. As in previous studies, it was found that NCS-1 fluorescence peaked at 340 nm in the absence of Ca^2+^ (see Figure 3 A). Increasing free Ca^2+^ up to 0.06 µM caused a slight increase in peak height, while its further increase led to a fall in peak height, and a shift in peak fluorescence to around 337 nm (see Figure 3A,B).

Peak NCS-1(R102Q) fluorescence did not fall to the same extent, but this may be because one of the only two tryptophan residues in NCS-1(R102Q), W103, is in a position adjacent to the mutated residue. Importantly, changes in wild-type and mutant NCS-1 fluorescence followed a similar pattern, suggesting that the proteins underwent conformational change at the same Ca^2+^ concentration (see Figure 3C).

### The R102Q mutation does not affect binding of NCS-1 to IL1RAPL1 In vitro

Interestingly, the C-terminal region identified by NMR as altered in NCS-1(R102Q) corresponds almost exactly to the minimal NCS-1(174–190) domain required for its binding to IL1RAPL1 [24]. Experiments were therefore carried out to determine whether the mutation affected the interaction between NCS-1 and the NCS-1-binding domain of IL1RAPL1 in vitro. GST and GST-IL1RAPL1(569–644) were immobilised on glutathione-Sepharose beads, and incubated with recombinant NCS-1 or NCS-1(R102Q) in the presence of varying concentrations of free Ca^2+^ (Figure 3). Specific binding of NCS-1 and the mutant to GST-IL1RAPL1(569–644) was observed. In contrast to a previous report [24], interaction between GST-IL1RAPL1(569–644) and NCS-1 was only detected in the presence of Ca^2+^ and at similar Ca^2+^ concentrations to those where changes in tryptophan fluorescence occurred. However, in five separate experiments, no consistent differences between the binding of NCS-1 and NCS-1(R102Q) to GST-IL1RAPL1(569–644) were identified nor were any differences in Ca^2+^-dependency observed. In a separate experiment to quantify binding to GST-IL1RAPL1(569–644), the level of binding of NCS-1(R102Q) was no different (97.3±4.9%, n = 4) from that for wild-type NCS-1 at 1 µM free Ca^2+^ concentration after correction for levels of input protein (Figure S1).

### Exogenous NCS-1 and NCS-1(102Q) localise identically in N2A cells, and partially colocalise with exogenous IL1RAPL1

Since no functional differences between NCS-1 and NCS-1(R102Q) proteins were identified *In vitro*, it was decided to examine their expression, localisation and characteristics in cells. Since the NMR data indicated that the R102Q mutation affected the IL1RAPL1-binding domain of NCS-1, and since we had not ruled out the possibility of a cellular effect on the NCS-1-IL1RAPL1 interaction, we chose to use cells that endogenously express both NCS-1 and IL1RAPL1 in this study. Previous studies on NCS-1 and IL1RAPL1 have been conducted using PC12 cells, but endogenous expression of IL1RAPL1 has not been detected in these cells [24], [26]. We therefore identified mouse N2A neuroblastoma cells as expressing both proteins (Figure S2), and characterised NCS-1 localisation in this cell line.

**Figure 4:**
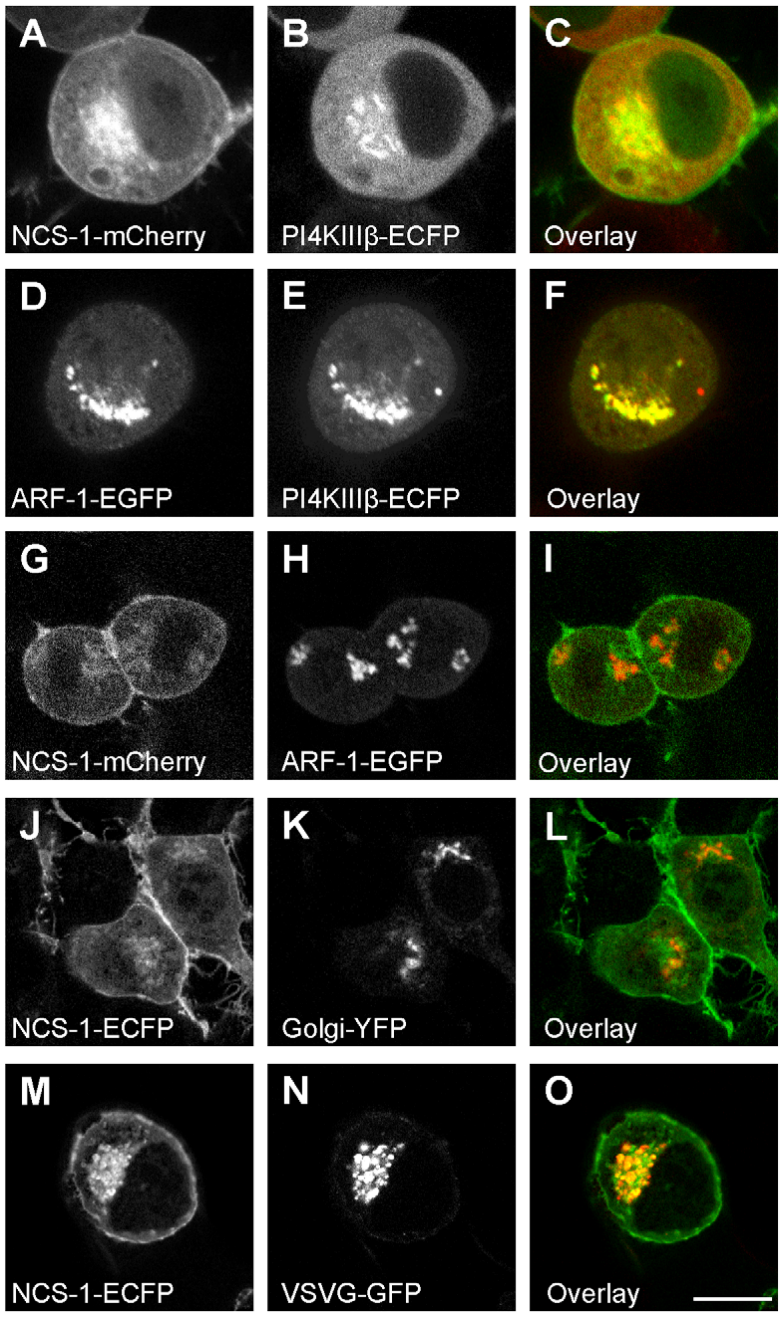
Localisation of exogenously expressed NCS-1 in N2A cells. N2A cells were transfected to express the indicated constructs and live cells were imaged using confocal microscopy. For the colour overlays (C,F,I,L,O) the images in A,D,G,J and M are in green and those in B,E,H,K,N are in red with co-localisation appearing in yellow. All images were taked form live cells except for those expressing VSVG-GFP which were fixed after transfection and incubation overnight at The scale bars represent 10 µm.

NCS-1 associates with the plasma membrane and Golgi complex as a result of its N-terminal myristoylation [47], [48]. Exogenously expressed fusion proteins of NCS-1, containing a C-terminal GFP-variant moiety, correctly localise to the Golgi complex and plasma membrane in various cell types, and appear to colocalise at the Golgi with known NCS-1 binding partners including ARF-1 and PI4KIIIβ [49], [50], [51], [52]. However, when we compared the localisation of NCS-1-mCherry to that of PI4KIIIβ-ECFP in N2A cells, we identified only partial colocalisation in the perinuclear region (Figure 4, A–C). It has been suggested that NCS-1 and the small GTPase ARF-1 might compete in recruitment of PI4KIIIβ to the Golgi [49]. Supporting this view, we found that ARF-1-EGFP colocalised with PI4K-ECFP (Figure 4 D–F) but ARF-1-EGFP poorly with NCS-1-mCherry (see Figure 4, G–I). Our interpretation of these data is that NCS-1 and its binding partners adopt discrete but overlapping localisations, and that NCS-1 localises to a specific Golgi subdomain in N2A cells. To characterise the localisation of NCS-1 at the Golgi complex, NCS-1-ECFP was compared to two Golgi markers. In live cell experiments, it was found that NCS-1-ECFP was colocalised poorly (Figure 4 J–L) with a Golgi-YFP (1,4-galctosyltransferase(1-81)-YFP) which is targeted to the cis-Golgi. To label the trans-Golgi network (TGN), we transfected cells with a fluorescent variant of the viral cargo protein VSVG, and used a 20°C temperature-block to prevent its exit from the TGN. Cotransfected NCS-1-ECFP colocalised well with this protein at the Golgi (Figure 4, M–O), suggesting that it associated with the membranes of the TGN.

**Figure 5:**
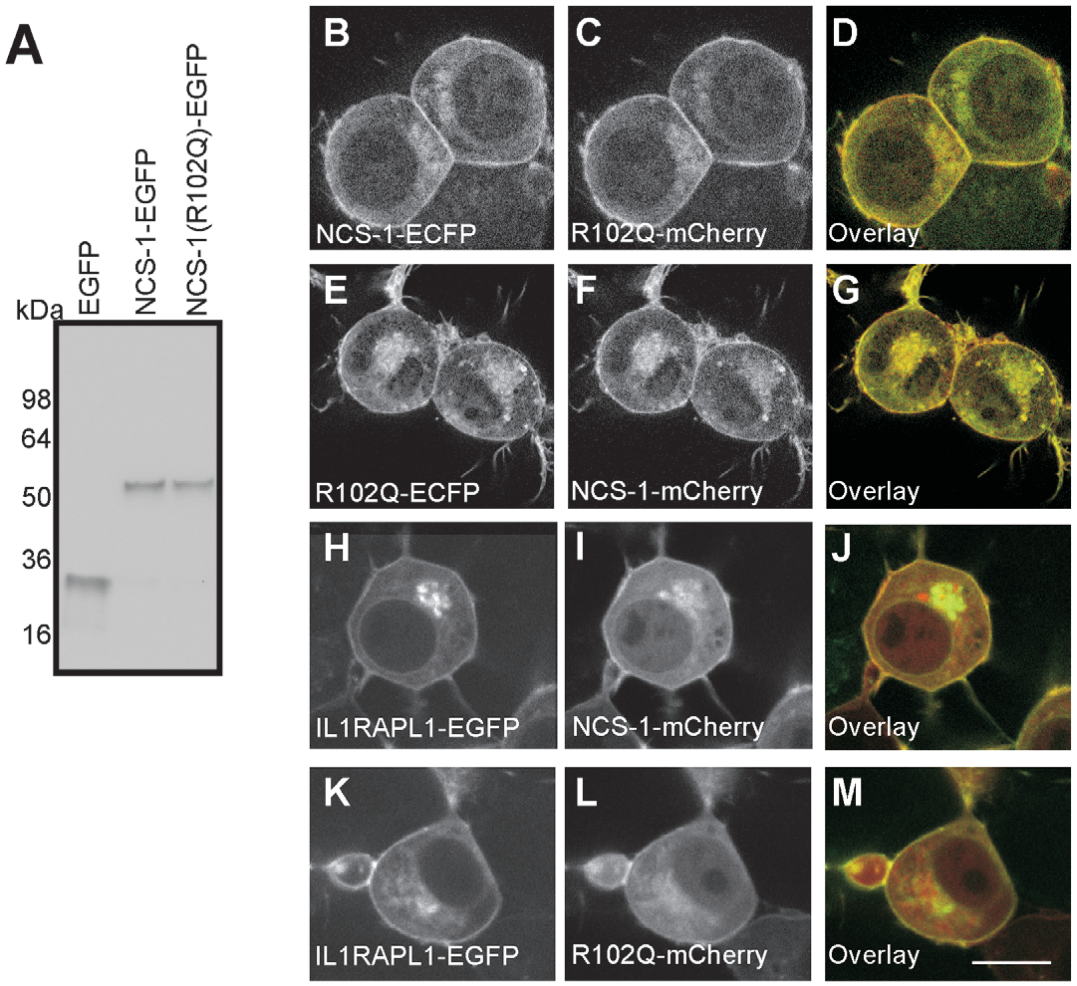
NCS-1 and NCS-1(R102Q) are similarly expressed and localised in N2A cells. N2A cells were transfected to express the indicated constructs and live cells were imaged using confocal microscopy. (A) Western blot of cells with anti-GFP showing protein expressed from a control EGFP vector, or plasmids encoding NCS-1-EGFP or NCS-1(R102Q)-EGFP. (B–M) For the colour overlays (D,G,J,M) the images in B, E, H, and K are in green and those in C,F, I, and L are in red with co-localisation appearing in yellow. The scale bars represent 10 µm.

To identify any difference in the localisation of NCS-1 and NCS-1(R102Q), N2A cells were alternately cotransfected with NCS-1-ECFP and NCS-1(R102Q)-mCherry, and NCS-1(R102Q)-ECFP and NCS-1-mCherry. When the lysates from individually transfected wells were subjected to SDS-PAGE and Western blotting, and the blots were probed with an antibody against GFP, it was found that the proteins were expressed at the same levels (Figure 5A) indicating no differences in the stability of the two proteins. When coexpressing cells were imaged, the NCS-1 and NCS-1(R102Q) fusions were found in the same localisations at the plasma membrane and the TGN of cells (Figure 5, B–D and E–G). We also compared the localisation of NCS-1 and NCS-1(R102Q)-mCherry with IL1RAPL1-GFP. The co-localisation between NCS-1 and NCS-1(R102Q)-mCherry in the perinuclear region was partial but both colocalised similarly with IL1RAPL1-GFP at the plasma membrane (see Figure 5 H–J and K–M).

### Over-expression of NCS-1 and NCS-1(R102Q) similarly affects Ca^2+^ signalling in N2A cells

**Figure 6:**
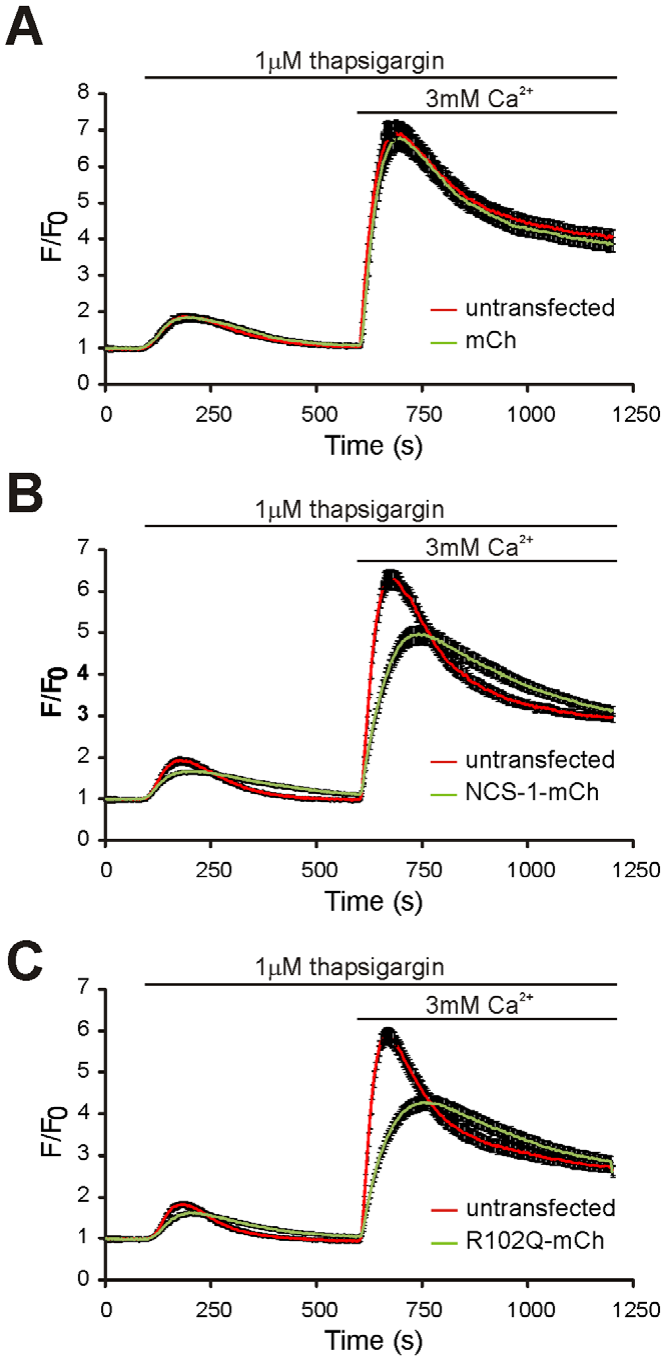
Expression of NCS-1 or NCS-1(R102Q) has similar inhibitory effects on intracellular Ca^2+^ signals in N2A cells. N2A cells were transfected to express mCherry from a control vector (A), NCS-1-mCherry (B) or NCS-1(R102Q)-mCherry (C) and loaded with the Ca^2+^ indicator Fluo4. Changes in Fluo4 fluorescence were monitored over time following addition of 1 µM thapsigargin in the absence of extracellular Ca^2+^ and subsequent addition of 3 mM Ca^2+^ to the external medium. Fluorescence values were expressed as a ratio of the starting fluorescence value for each cell and are shown as mean ± SEM (n = 49−61 cells per condition). In each experiment Fluo4 fluorescence was monitored in transfected and adjacent untransfected cells in the same microscope fields for direct comparison.

As we had identified no deficits in NCS-1(R102Q) calcium-binding *in vitro*, or in its localisation in N2A cells, we proceeded to examine whether the mutation might affect the capacity of NCS-1 to modulate intracellular Ca^2+^ signalling. The involvement of NCS-1 in Ca^2+^ signalling is complex, as it has been suggested to act via various target proteins. It has been reported to interact with InsP3 receptors, and to enhance their activity in a Ca^2+^-dependent manner [53]. It has also been reported to either positively or negatively regulate P/Q-type voltage-gated Ca^2+^ channels (VGCCs), and to be required for the negative regulation of N-type channels by IL1RAPL1 [26], [54] [55]. Furthermore, NCS-1 may regulate channels either directly, or by influencing phosphoinositide signalling or membrane trafficking [49], [56]. In order to raise intracellular Ca^2+^ in the present study, we used the SERCA pump inhibitor thapsigargin to release Ca^2+^ from the endoplasmic reticulum followed by Ca^2+^ addition to the external medium to determine general effects of NCS-1 over-expression on Ca^2+^ release and entry. N2A cells were examined in a protocol in which they were exposed to 1 µM thapsigargin in the absence of extracellular Ca^2+^ in order to release calcium from internal stores, and then extracellular Ca^2+^ was added to a concentration of 3 mM in order to provoke store-operated calcium entry. The resulting changes in intracellular Ca^2+^ concentration were monitored using the Ca^2+^ indicator Fluo-4, and the responses of untransfected and transfected cells in the same dishes were directly compared. No significant difference was identified between the responses of untransfected cells and cells transfected to express mCherry as a control, and in both cases, there was an initial increase in intracellular Ca^2+^ concentration after addition of thapsigargin, and this was followed by a larger rise in intracellular Ca^2+^ concentration after addition of extracellular Ca^2+^ (Figure 6A). In contrast, when the responses of cells transfected to express either NCS-1-mCherry or NCS-1(R102Q)-mCherry were compared to those of untransfected controls, differences were observed (Figure 6). Expression of NCS-1-mCherry or NCS-1(R102Q)-mCherry led to a reduction in the rate of increase in Ca^2+^ levels following thapsigargin treatment and also following addition of extracellular Ca^2+^ (Figure 6B,C). There was, however, no significant difference in the overall changes in intracellular Ca^2+^ concentration between cells expressing each of the two NCS-1 constructs.

### NCS-1 and NCS-1(R102Q) associate dynamically with cellular membranes

**Figure 7:**
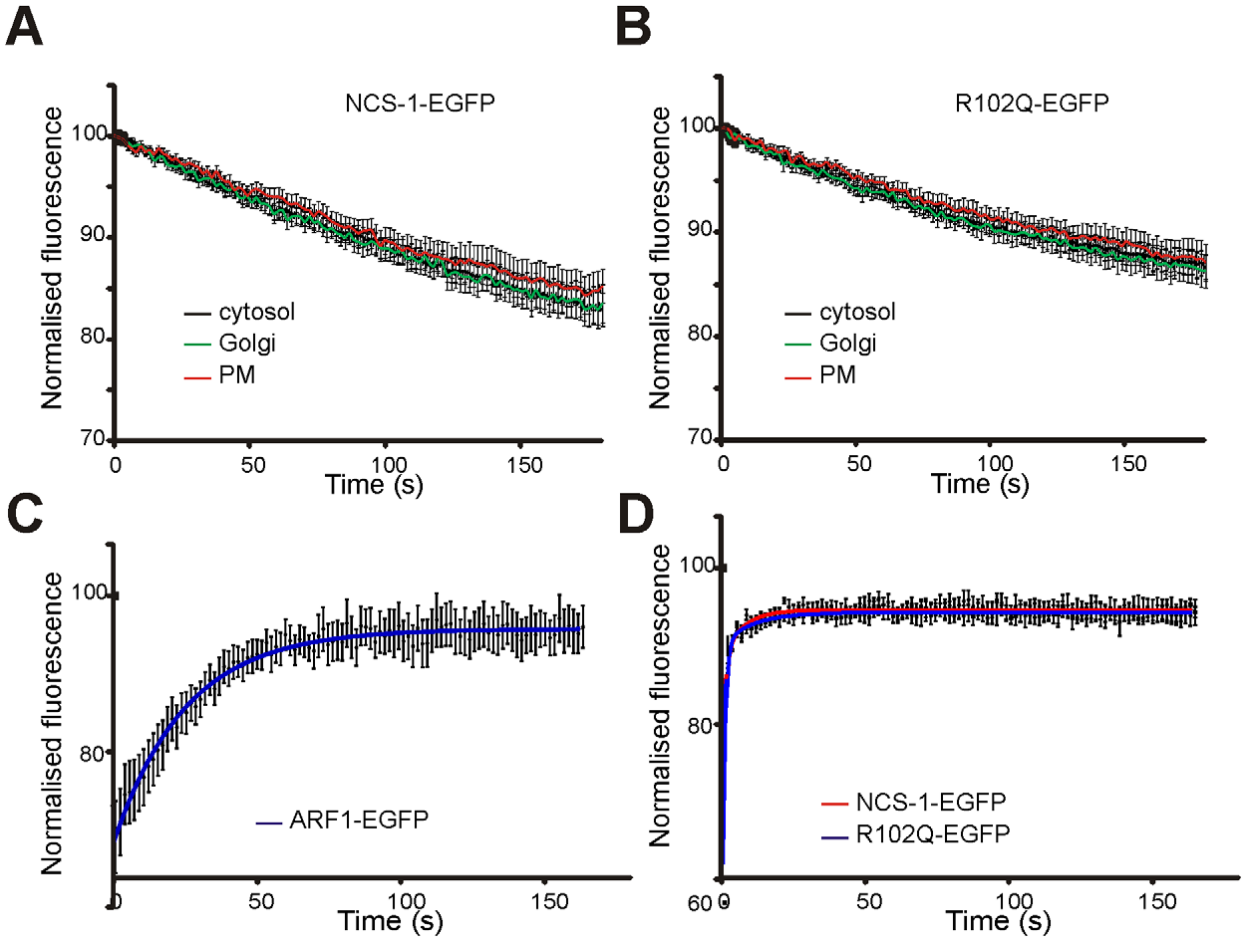
NCS-1 and NCS-1(R102Q) associate dynamically with the plasma membrane and TGN in N2A cells. (A) Cells transfected to express NCS-1-EGFP were treated in a FLIP protocol in which a small region of the cytosol was repetitively photobleached and fluorescence was monitored in regions of the cytosol, plasma membrane and Golgi complex. Data are shown as mean ± SEM (n = 8 cells). Fluorescence declined in all three regions with the same time course. (B) Cells transfected to express NCS-1(R102Q) -EGFP were treated in the FLIP protocol. Data are shown as mean ± SEM (n = 14 cells). Fluorescence declined in all three regions with the same time course. (C) Cells transfected to express ARF1-EGFP were subjected to a FRAP protocol in which a region of interest over the Golgi complex was photobleached and the recovery of fluorescence in the same region was monitored over time. Data are shown as mean ± SEM (n = 9 cells). The curve shows the data fitted using non-linear regression to one phase recovery kinetics. (D) Cells transfected to express NCS-1-EGFP or NCS-1(R102Q)-EGFP were subjected to a FRAP protocol in which a region of interest over the Golgi was photobleached and the recovery of fluorescence in the same region was monitored over time. Data are shown as mean ± SEM (n = 12 cells per condition). The curves shows the data fitted using non-linear regression to two phase recovery kinetics.

Previous work has shown that unlike the situation with a number of other NCS family members, the myristoyl group of NCS-1 is continually exposed, and NCS-1 is membrane-associated, at resting Ca^2+^ concentrations [47]. However, it has also been reported that ∼30% of cellular NCS-1 is cytosolic [16], [57]. This raises the possibility that NCS-1 cycles between membrane-associated and cytosolic states even at resting Ca^2+^ concentrations, and only interacts with membranes transiently. This possibility has not, however, been investigated. In order to establish whether NCS-1 associates with membranes in a transient or a stable manner, fluorescence loss in photobleaching (FLIP) experiments were carried out. The rationale behind this technique is that where a partially cytosolic fluorescent protein associates transiently with membranes, progressive bleaching of the cytosolic pool leads to parallel loss of cytosolic and membrane fluorescence. Whereas, when a protein is more stably associated with membranes, membrane fluorescence is lost at a much slower rate. Parts A and B of Figure 7 show FLIP data from cells expressing EGFP-labelled wild-type or NCS-1(R102Q). The aim of this experiment was primarily to compare the bleaching times for cytosol, Golgi and plasma membrane for each protein. The cells expressing wild type and mutant proteins were analysed on different days and so the bleaching rates for each protein cannot be directly compared between Figure 7A and 7B. As seen in Figure 7, repetitive bleaching of either NCS-1- or NCS-1(R102Q)-EGFP within a region of the cytosol led to loss of Golgi- and plasma membrane-associated fluorescence that paralleled the loss of cytosolic fluorescence in each case. These data indicate that these proteins must interact with membranes transiently and rapidly exchange between cytosolic and membrane pools.

To compare the dynamics of NCS-1- and NCS-1(R102Q)-membrane interactions at the TGN in more detail, fluorescence recovery after photobleaching (FRAP) experiments were conducted. The small GTPase ARF-1, which has also been shown to interact with Golgi membranes in a transient manner [58] was used as a positive control in these experiments. In a FRAP experiment, the fluorescent protein within a small region of interest (ROI) of a cell is bleached using a high-intensity laser, and the recovery of fluorescence within this area is subsequently monitored [59]. The recorded fluorescence recovery reflects the replacement of bleached protein in the ROI with unbleached protein from outside this region. The data in Figure 7, C and D, derives from the bleaching of circular regions of the Golgi of 2 µM diameter and monitoring of the fluorescence recovery. ARF-1-EGFP recovery was best fit by one-phase association kinetics with its recovery reaching a plateau at 95.97±0.14% and a recovery halftime of 17.29s. Fluorescence recovery of NCS-1-EGFP and NCS-1(R102Q)-EGFP was rapid and almost complete, and their recovery profiles were nearly superimposable. Non-linear regression analysis indicated that recovery was better fit by two-phase than by one-phase association kinetics (p<0.0001), and that recovery reached a plateau at 98.13±0.06 and 97.79±0.09%, with halftimes calculated as 0.55 s and 4.65 s, and 0.58 s and 7.19 s, for NCS-1 and NCS-1(R102Q) respectively. Taken together these data from both FLIP and FRAP experiments suggest that both the wild type and mutant NCS-1 proteins interact with membranes transiently, and if this interpretation is correct then it appears that they cycle on and off membranes more rapidly than ARF-1.

### Ca^2+^ elevation differentially affects NCS-1 and NCS-1(R102Q) dynamics at the plasma membrane

**Figure 8:**
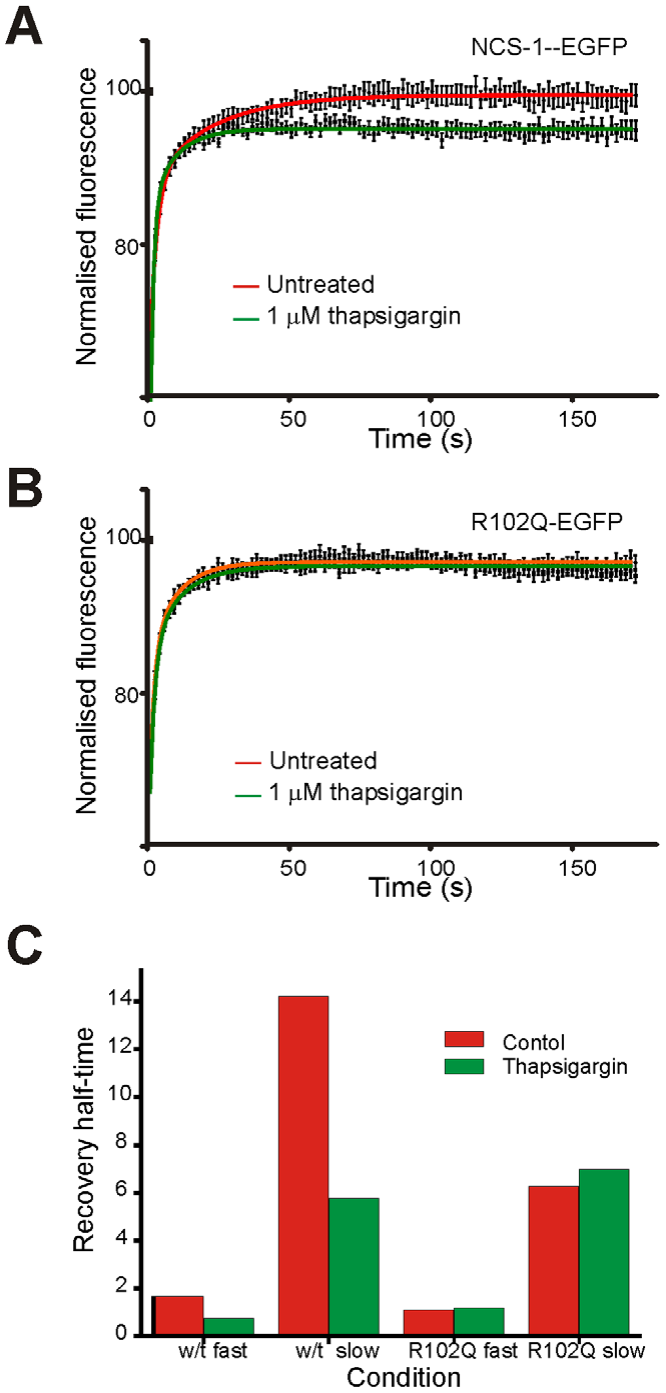
The dynamics of NCS-1 and NCS-1(R102Q) at the plasma membrane in N2A cells and the effects of elevation of intracellular Ca^2+^ differ. (A) Cells transfected to express NCS-1-EGFP were subjected to a FRAP protocol in which a region of interest over the plasma membrane was photobleached and the recovery of fluorescence in the same region was monitored over time. Cells were either untreated or were exposed to 1 µM thapsigargin in the presence of 3 mM external Ca^2+^. Data are shown as mean ± SEM (n = 34 and n = 36 for control and thapsigargin-treated cells respectively. The curves shows the data fitted using non-linear regression to two phase recovery kinetics. (B) Cells transfected to express NCS-1(R102Q)-EGFP were subjected to a FRAP protocol in which a region of interest over the plasma membrane was photobleached and the recovery of fluorescence in the same region was monitored over time. Cells were either untreated or were exposed to 1 µM thapsigargin in the presence of 3 mM external Ca^2+^. Data are shown as mean ± SEM (n = 38 and n = 40 for control and thapsigargin-treated cells respectively. The curves shows the data fitted using non-linear regression to two phase recovery kinetics. (C) Values for the calculated half-times of recovery from a non-linear regression analysis based on two-phase association kinetics.

It was important to continue characterisation of the association/dissociation kinetics of NCS-1 and NCS-1(R102Q), as although the kinetics were no different at the TGN, some of the functions of NCS-1 are restricted to the plasma membrane. FRAP was used to assess the dynamics of NCS-1 and NCS-1(R102Q)-EGFP. In these experiments, cells expressing each of the proteins were tested on the same day with measurements alternating between wild-type and mutant expressing cells so that the data for each protein could be directly compared. Upon examination of plasma membrane association/dissociation kinetics, it was found that the recovery of NCS-1-EGFP and NCS-1(R102Q)-EGFP fluorescence differed (Figure 8 A,B).

Under resting conditions, recovery was fast with both proteins and best fit by two-phase association kinetics, but while NCS-1-EGFP fluorescence recovered with halftimes of 1.64 s and 14.23 s, NCS-1(R102Q)-EGFP fluorescence recovered with halftimes of 1.11 s and 6.27 s (Figure 8C). While the recovery of NCS-1-EGFP plateaued at 98.85±0.06% that of NCS-1(R102Q)-EGFP plateaued significantly lower at 96.2%±0.07 (Figure 8). In combination, these data suggest that a portion of NCS-1(R102Q)-EGFP is not replaced by unbleached protein following photobleaching, but is retained in the bleached region.

Reports conflict as to whether calcium elevation causes NCS-1 translocation to the plasma membrane. While no change was identified in NCS-1 localisation by conventional confocal microscopy upon an increase in intracellular calcium [47], NCS-1 is more strongly associated with membrane fractions prepared in the presence of calcium than with fractions prepared in its absence [16], [57]. This discrepancy may be explained, if NCS-1 is transiently membrane associated under resting conditions, but a fraction of the protein becomes more stably associated on Ca^2+^ elevation. In order to test this hypothesis, we conducted parallel bleaching experiments on cells transfected to express the NCS-1 constructs in the presence or absence of 1 µM thapsigargin. We used this reagent to raise intracellular Ca^2+^ since we had already demonstrated that it produces comparable, long-lasting calcium elevations in cells expressing NCS-1-EGFP or NCS-1(R102Q)-EGFP (Figure 7). As compared to the calculated association kinetics in untreated cells expressing NCS-1-EGFP, we found that in the presence of thapsigargin, the fast phase of association was not significantly altered, but that the slow phase of association was affected (Figure 8A,C). Furthermore, fluorescence recovery reached plateau at a lower level in the treated cells (94.6±0.06% compared to 98.85±0.0.07%). In contrast we found that thapsigargin treatment had no significant effect on the fluorescence recovery profile of NCS-1(R102Q)-EGFP (Figure 8B). We conclude that while wild-type NCS-1 is stabilised at the plasma membrane upon elevation of intracellular Ca^2+^ concentration, the R102Q mutant protein is already partly deficient in its capacity to dissociate from the plasma membrane under resting conditions and so elevated Ca^2+^ does not affect its dynamics.

## Discussion

The data presented in this paper provide insights into the cellular localisation and dynamics of NCS-1 and describe structural and functional deficits in the R102Q mutation/polymorphism of NCS-1 that had been discovered in an individual with ASD [34]. We used NMR spectroscopy to generate and compare the ^1^H/^15^N HSQC spectra of recombinant NCS-1 and NCS-1(R102Q). The ^1^H/^15^N HSQC spectrum of the mutant protein showed a number of resonances from residues towards the C-terminus which are either absent or significantly reduced in intensities when compared to the spectrum of the wild-type protein. The mutation of this single surface residue appeared to have produced pronounced, long-range as well as local changes. It is interesting to note that while R102 is not conserved in lower organisms such as *C. elegans* and *Drosophila*, it is conserved in all species from fish to man. The AGADIR analysis predicts a reduction in helical content in helix F when R102 is mutated to a glutamine. Therefore, it is most likely that the destabilisation of helix F affects the hydrogen-bond network which in the wild-type protein restraints the conformational dynamics of the C-terminus region. A result of this hydrogen bond perturbation is that the C-terminus is able to adopt several conformations which are in chemical exchange. Despite these structural changes in NCS-1(R102Q), the protein shows a Ca^2+^-dependent conformation change as monitored by tryptophan fluorescence at the same Ca^2+^ concentration as wild type protein, localised essentially identically to wild-type NCS-1 when expressed in N2A cells and had a similar effect on thapsigargin-induced Ca^2+^ signals.

The localisation of exogenous NCS-1 in N2A cells that we identified in this study is similar to that seen in other cell types in previous studies [47], [49], [50], [51], [52]. However, the localisation of NCS-1 with respect to the Golgi complex had not previously been examined in detail, and in this study we show that NCS-1 preferentially associates with a subdomain of the Golgi, the TGN, but not with cis-regions of the Golgi. Previous work, based on steady-state imaging, was interpreted as showing that a substantial proportion of NCS-1 was stably membrane-associated through its N-terminal myristoyl tail in resting cells 1[47]. However, other partly cytosolic peripheral proteins, such as GTP-binding proteins [58], [60], [61], are known to cycle between membranes and cytosol, and an increase in NCS-1 membrane association is reported in response to cellular Ca^2+^ elevation [57], [62]. The dynamics of cycling can be assessed using analysis of FRAP. Evidence of recovery of membrane fluorescence in FRAP experiments could be due to either exchange of the fluorescent protein between membrane and cytosolic pools or to rapid mobility of the protein in the membrane. We have found using FLIP, however, that photobleaching of the cytosol resulted in a parallel loss of NCS-1- and NCS-1(R102Q)-EGFP fluorescence on the Golgi and plasma membranes. This finding can only be explained by rapid cycling between membrane and cytosolic pools and suggests that the association of NCS-1 and R102Q with the TGN and the plasma membrane is highly dynamic under resting conditions. From FRAP experiments, it was observed that Ca^2+^ elevation stabilises a fraction of cellular NCS-1 at the plasma membrane. Presumably, this stabilisation follows Ca^2+^ -binding by NCS-1 and possibly Ca^2+^-dependent interaction with a binding partner. It has been previously shown that bradykinin stimulation, which raises intracellular Ca^2+^ via IP_3_ -induced calcium release, can shift a proportion of cellular NCS-1 into detergent resistant plasma membrane microdomains [63] and it is possible that a calcium-dependent protein-protein interaction drives this relocation.

While the major structural changes in NCS-1(R102Q) were in the domain required for binding to IL1RAPL1, the main functional effect of the mutation was loss of a Ca^2+^-dependent component of cytosol/plasma membrane exchange dynamics. Thus, one possibility is that in cells, wild type NCS-1 binds to an endogenous plasma membrane target protein effectively only when Ca^2+^ is elevated, but NCS-1(R102Q) is able to bind to the target protein via its C-terminus at resting Ca^2+^ concentrations. Assay of in vitro binding of NCS-1 and NCS-1(R102Q) to GST-IL1RAPL1(569–644) did not reveal any differences, but this may not be surprising, as NCS-1 does not need to be fully folded to bind to IL1RAPL1. Indeed, the C-terminus alone, NCS-1(174–190), is sufficient for an interaction [24]. Use of a C-terminal fragment of NCS-1 as an inhibitory reagent in cellular studies has suggested the existence of other important functional interactions through this region of the protein [7], [17], [55], but so far only IL1RAPL1 has been shown to bind to this region of NCS-1 directly.

The functional significance of NCS-1 dynamics and its Ca^2+^-dependent stabilisation at the plasma membrane are not yet clear, but it is tempting to speculate that the dynamic interaction between NCS-1 and membranes might allow regulation of its local levels on neuronal membranes. Impairment of this regulation could have subtle effects on neuronal signalling and physiology in developing and adult brain. It is noteworthy that NCS-1 is highly conserved having an identical amino acid sequence in birds and all mammals. There are, for example, only four amino acid differences between NCS-1 in humans and the most similar NCS-1 in the teleost fish *Danio rerio* suggesting strong evolutionary pressure against any changes in its sequence. There is currently little evidence available for non-synonomous polymorphisms in NCS-1 in human populations. A direct linkage of the R102Q mutation/polymorphism to ASD has not yet been established as it is based on data from a single subject. Further genetic and functional analysis may now be warranted, however, to determine the relevance of the R102Q mutation/polymorphism,in NCS-1 to the pathology of ASD.

## Materials and Methods

### Plasmids

The NCS-1 sequence was excised from an existing vector [47] and inserted into pECFP-N1, pEGFP-N1and pmCherry-N1 vectors (Clontech, Basingstoke, UK) to generate mammalian expression constructs for NCS-1, C-terminally tagged with variant fluorescent proteins. The fragment encoding NCS-1, and the target vectors, were generated by digestion with *Bam*HI/*Xho*I and were ligated using standard methods. The vector used for the bacterial expression of GST-NCS-1 was as described previously [64]. With the above plasmids as templates, NCS-1(R102Q) plasmids were made by substitution of the 102^nd^ codon, AGG, for CAG, using the primer pair 5′-GGACCCTGGATGAGAAGTTGCAGTGGGCCTTC-3′ (sense) and 5′-CAACTTCTCATCCAGGGTCCCCCTTGAGG-3′ (antisense). Mutants were generated using the GeneTailor system (Invitrogen, Paisley, UK) according to manufacturer's instructions. All constructs were verified by automated sequencing (The Sequencing Service, Dundee, UK). The Golgi-YFP fusion construct was obtained from Clontech, and the pIL1RAPL1-GFP [24], pARF1-EGFP [49], pVSVG-GFP [65], pPI4KIIIβ-ECFP [50] and pFAPP1-EGFP [66] fusion constructs were as described previously. The plasmid encoding GST-IL1RAPL1(569–644) was prepared by amplification of the appropriate region from pILRAPL1-GFP and ligation into pGex-6P-1 (Amersham Biosciences).

### Recombinant protein production

GST, GST-IL1RAPL1(569–644), GST-NCS-1 and GST-NCS-1(R102Q) were expressed in BL21 E.coli. Unlabeled protein was produced by inoculating SB media, supplemented with 100 mg/l ampicillin, with single transformed clones, allowing cell growth at 37°C for 15–17 h, and inducing protein expression with 1 mM isopropyl-β-d-thiogalactopyranoside (IPTG) for 3–4 h. ^15^N-labeled GST-NCS-1 and GST-NCS-1(R102Q) were produced by similar inoculation of M9 minimal media, supplemented with 100 mg/l ampicillin and containing (^15^NH_4_)SO_4_ as a sole nitrogen source with growth at 37°C to a density of 0.8 OD units and induction with 1 mM IPTG overnight at 30°C. Following cell disruption by use of a French press, recombinant proteins were purified on glutathione-conjugated Sepharose. GST tags were removed from NCS proteins using PreScission protease (Invitrogen) according to the manufacturer's instructions prior to use in all experiments.

### NMR spectroscopy

The NMR samples of NCS-1 and NCS-1(R102Q) were approximately 0.5 mM in 20 mM Tris, 50 mM NaCl, 5 mM MgCl_2_, 10 mm CaCl_2_, 1 mM NaN_3_ in 90% H_2_O/10% D_2_O at pH 6.5. Spectra were recorded at 30°C using a Bruker Avance 800 MHz spectrometer fitted with a triple resonance, single z-axis gradient cryogenic probehead. ^15^N-^1^H spectra were acquired using water flip-back pulses. Spectra were processed using Bruker Topspin software.

### Binding assays

GST or GST-IL1RAPL1(569–644) was bound to 15 µl glutathione-Sepharose beads for 1 h at 4°C. Beads were washed with binding buffer (139 mM NaCl, 20 mM HEPES, 5 mM EGTA, 5 mM nitrolotriacetic acid, 6.44 mM MgCl_2_, 1 mM DTT, pH 7.4) containing CaCl_2_ to give free calcium concentrations of 0, 0.1, 0.3 or 1 µM, before incubation with 1 µM recombinant NCS-1 or NCS-1(R102Q) for 1 h at 4°C. The beads were washed three times in the appropriate buffer, and then bound proteins were eluted by boiling in SDS/PAGE sample buffer. Samples were separated by SDS PAGE and proteins were stained using Coomassie Brilliant Blue. To allow quantification of binding to IL1RAPL1, multiple binding assays were carried out at 1 µM free Ca^2+^ concentration and samples of bound proteins and input proteins run on the same gels. Quantification of bound NCS-1 protein was carried out using ImageJ (NIH, Bethesda, Maryland, USA) and values corrected for concentration of the input proteins.

### Spectrofluorometry

To monitor intrinsic tryptophan fluorescence of NCS proteins [43], purified recombinant NCS-1 or NCS-1(R102Q) at a concentration of 1 µM in a calcium free buffer (25 mM Tris HCl, 50 mM KCl, 5 mM EGTA, 5 mM nitrolotriacetic acid, 6.44 mM MgCl_2_ (2 mM free Mg^2+^),1 mM DTT, pH 7.4) were excited at room temperature with 280 nm light, and their emission spectra from 290–410 nm were measured, using a Jasco FP-6300 spectrofluorometer (Tokyo, Japan). The concentration of free calcium was increased by incremental addition of CaCl_2_ to give calculated free Ca^2+^ concentrations, and additional emission spectra were measured as before.

### Cell culture and transfection

Adherent N2A cells (American Type Culture Collection. Manassas, Virginia, USA) were grown in 75 cm^2^ culture flasks at 37°C, 5% CO_2_ in Dulbecco's Modified Eagle Medium (Gibco, Paisley, UK) supplemented with 10% foetal calf serum, 100 U/ml penicillin and 0.1 mg/ml streptomycin. Cells were passaged twice weekly. Transfection of the cells was carried out as follows. Coverslips in live cell dishes (MatTek, MA, USA) or in 6- or 24-well plates were seeded with ∼3×10^5^ cells/ml media, and cells were allowed to adhere overnight. Transfection mixes were made up with 3 µl of the Gene Juice reagent (Novagen, Prudhoe, UK) per µg plasmid in 100 µl Optimem 1 (Gibco), incubated for 30 minutes, and then applied to cells. Cells were incubated for a further 18 hours prior to experiments. Cells expressing VSVG-GFP were incubated for 18 h at 20°C and then washed in PBS and fixed in 4% (v/v) paraformaldehyde for 30 min prior to imaging. All other imaging was carried out on live cells.

### Western blotting

Cell lysates were made by direct addition of 200 µl/well Laemmlli buffer (Sigma) to cells in 6-well plates following washing with PBS. Samples were resolved using SDS-PAGE (12.5% gels) and transferred to nitrocellulose filters for Western blotting by transverse electrophoresis. Anti-NCS-1 (Transduction Laboratories, Oxford, UK), anti-IL1RAPL1 (AbCam, Cambridge, UK) and anti-GFP (JL-8) (Clontech) were used at 1∶800, 1∶200 and 1∶1000 dilutions respectively. HRP-conjugated secondary antibodies (Sigma) were used at a 1∶400 dilution, and blots were analysed by detection of chemiluminescence using a Biorad Chemidoc XRS system (Biorad, Hemel Hempstead, UK).

### Confocal fluorescence microscopy

Confocal microscopy following fixation, in live cell single-frame imaging and in calcium indicator experiments, was carried out on a Leica TCS-SP2 microscope (Leica Microsystems, Heidelberg, Germany) using a 63x oil-immersion objective with a 1.4 numerical aperture. The pinhole was set to airy2 for calcium indicator experiments and to airy1 for all other experiments. Fluorescence loss in photobleaching (FLIP) and fluorescence recovery after photobleaching (FRAP) experiments were carried out on a Leica TCS-SP2-MP microscope (Leica Microsystems). A 63x water-immersion objective with a 1.2 numerical aperture was used and the pinhole set to airy 2.06. Where variant GFP-containing constructs were used, wells/dishes were transfected with 0.5 µg of each plasmid. ECFP-, EGFP-, and mCherry-containing constructs were excited using 405 nm, 488 nm and 594 nm lasers respectively, and emitted light was collected between 450–500 nm, 500–550 nm and 625–675 nm. Fluo-4 was excited using a 488 nm laser, and emitted light was collected between 495–545 nm. Golgi-YFP was excited using a 514 nm laser, and emitted light was collected between 530–600 nm. All imaging of live cells and was carried out on cells at room temperature in Krebs Ringer buffer (145 mM NaCl, 20 mM HEPES, 10 mM Glucose, 5 mM KCl, 1.3 mM MgCl_2_, 1.2 mM NaH_2_PO_4_).

In calcium indicator experiments, cells were loaded with 5 µM Fluo-4-AM (Invitrogen) for 30 minutes at room temperature to allow measurement of changes in [Ca^2+^]_i_. Equal numbers of untransfected and transfected cells were analysed in each field, and following background-subtraction using Image J software (NIH), Fluo-4 fluorescence measurements for each cell were normalised with respect to fluorescence at the first time-point.

In FLIP experiments, rectangular regions of interest (ROIs), 4 µm by 2 µm, in the cytosol of cells, were repetitively bleached using 75% of laser power. To discriminate fluorescence loss in different cellular regions, background fluorescence was subtracted from images and ROIs drawn around the outside perimeter, inside perimeter, nucleus, and Golgi of cells. Values for average plasma membrane pixel intensity were calculated after subtracting fluorescence totals and pixel number contained by the inner perimeter from those contained by the outer perimeter. Values for average cytosol pixel intensity were calculated after subtracting fluorescence totals and pixel numbers contained by the nucleus, Golgi and bleached region from those contained by the inner perimeter. Data was normalised with respect to fluorescence of adjacent cells in each experiment in order to correct for bleaching during low power laser excitation.

In FRAP experiments, circular regions of interest of diameter 2 µm, were bleached using 75% of laser power following prior capture of 10 frames. 100 post-bleach frames were recorded per cell. To determine the rate of fluorescence recovery, fluorescence in these regions was measured over time, and then normalised with respect to corresponding total cellular fluorescence at each individual time point to correct for bleaching during low power laser excitation. Bleaching experiments with NCS-1-EGFP and NCS-1(R102Q)-EGFP, and experiments in the presence and absence of thapsigargin, were conducted in parallel. Non-linear regression analysis was used to calculate the rate and extent of fluorescence recovery of each construct and was carried out using Graphpad Prism software (Graphpad software, CA, USA).

## Supporting Information

Figure S1 Coomassie Blue-stained SDS-gel used for quantification of NCS-1 and NCS-1(R102Q) binding to GST-IL1RAPL1(569-644). Each gel has 4 independent binding assays on the left, carried out at 1 µM free Ca^2+^ concentration, and four lanes of the input NCS-1 protein on the right.
(5.82 MB TIF)

Figure S2 IL1RAPL1 and NCS-1 are endogenously expressed by N2A cells. A. Samples of N2A cells were separated by SDS-PAGE, prepared for Western blotting and probed with antisera specific for IL1RAPL1 or NCS-1 as indicated. B-D N2A cells were fixed and processed for immunufluorescence staining with anti-NCS-1 and anti-IL1RAPL1. The overlay shows anti-IL1RAPL1 in green and anti-NCS-1 in red with co-localisation in yellow in the overlay image.
(3.37 MB TIF)

Table S1 Propensity of helix formation predicted by use of Agadir (reference 48).
(0.09 MB TIF)
